# Does endometriosis increase susceptibility to COVID-19 infections? A case–control study in women of reproductive age

**DOI:** 10.1186/s12905-021-01270-z

**Published:** 2021-03-22

**Authors:** Bahram Moazzami, Shahla Chaichian, Saeed Samie, Masoumeh Majidi Zolbin, Fatemeh Jesmi, Meisam Akhlaghdoust, Mahin Ahmadi Pishkuhi, Zahra Sadat Mirshafiei, Fereshteh Khalilzadeh, Dorsa Safari

**Affiliations:** 1 Pars Advanced and Minimally Invasive Medical Manners Research Center, Pars Hospital, Tehran, Iran; 2 Pediatric Urology and Regenerative Medicine Research Center, Section of Tissue Engineering and Stem Cells Therapy, Children’s Hospital Medical Center, Tehran University of Medical Sciences, Tehran, Iran; 3 PhD Student in Epidemiology, Department of Epidemiology and Biostatistics, School of Public Health, Tehran University of Medical Sciences, Tehran, Iran

**Keywords:** Endometriosis, Coronavirus, COVID-19, SARS-CoV-2, Risk factors, Immunologic factors

## Abstract

**Background:**

In today’s world, coronavirus disease 2019 (COVID-19) is the most critical health problem and research is continued on studying the associated factors. But it is not clear whether endometriosis increases the risk of COVID-19.

**Methods:**

Women who referred to the gynecology clinic were evaluated and 507 women with endometriosis (case group) were compared with 520 women without endometriosis (control group). COVID-19 infection, symptoms, exposure, hospitalization, isolation, H1N1 infection and vaccination, and past medical history of the participants were recorded and compared between the groups using IBM SPSS Statistics for Windows version 21.

**Results:**

Comparison between the groups represent COVID-19 infection in 3.2% of the case group and 3% of the control group (*P* = 0.942). The control group had a higher frequency of asymptomatic infection (95.7% vs. 94.5%; *P* < 0.001) and fever (1.6% vs. 0%; *P* = 0.004), while the frequency of rare symptoms was more common in the case group (*P* < 0.001). The average disease period was 14 days in both groups (*P* = 0.694). COVID-19 infection was correlated with close contact (r = 0.331; *P* < 0.001 in the case group and r = 0.244; *P* < 0.001 in the control group), but not with the history of thyroid disorders, H1N1 vaccination, traveling to high-risk areas, and social isolation (*P* > 0.05).

**Conclusion:**

Endometriosis does not increase the susceptibility to COVID-19 infections, but alters the manifestation of the disease. The prevalence of the disease may depend on the interaction between the virus and the individual’s immune system but further studies are required in this regard.

## Background

Coronavirus disease 2019 (COVID-19) is one of the most critical pandemics ever, resulting in about 15% mortality rate in hospitalized patients [1]. As a newly emerging disease, ongoing research is running on different aspects of the disease [2]. The virus mainly affects the respiratory system, presenting with cough, difficult breathing, pneumonia, and in severe cases, results in acute respiratory distress syndrome (ARDS), need for intensive care unit (ICU) admission, and mechanical ventilation [3]. Some cases may even be complicated by multiple organ failure (MOF) which results in death [4].

Susceptibility of specific organs to COVID-19 has provoked research towards the disease mechanisms [5], which resulted in identification of spike glycoprotein (S protein), one of the main structural components of SARS-CoV-2, which facilitates binding of the envelope viruses to host cells by forming homotrimers protruding on the viral surface, which attracts angiotensin-converting enzyme 2 (ACE_2_) [6]. Therefore, SARS-CoV-2 can directly damage organs that express ACE_2_, including lungs, heart, kidneys, and intestines [7]; accordingly, the virus entry through this receptor depends on the cleavage of the S protein, which varies in different virus strains and cell types [8]. ACE_2_ protein is also effective in the physiology and pathology of the female reproductive system and fertility processes through production of sex hormones [9, 10], which is also affected during COVID-19 infection [11]. As suggested, the renin-angiotensin system (RAS) in ovaries and angiotensin (Ang)-II and Ang [1–7] facilitate follicle development, steroid secretion, oocyte maturation, and follicle atresia, as well as steroidogenesis and ovulation [12, 13], which justifies the association of ovarian ACE_2_ malfunction with reproductive disorders, like polycystic ovary syndrome and ovarian hyperstimulation syndrome [14], uterine leiomyoma [15], as well as endometriotic lesions [16, 17]. The expression of ACE_2_, the SARS-CoV cellular receptor, has been also detected in the endometrial tissue [15, 18]. Although the endometrium has an overall low risk of COVID-19 infection, due to the low expression of ACE_2_ and transmembrane protease serine protease-2 (TMPRSS2), but the expression of these host receptors increase at specific stages of the menstrual cycle and varies based on the woman’s age and endometrial susceptibility to the infection [19]. Previous studies on the endometrial disease have also determined the presence of ACE_2_ in the glandular epithelium, stroma, perivascular space, and endothelium [20, 21]. However, as the only study on the gene expression of SARS-CoV states, because of several limitations such as the small sample size, variability in the genetic profile of individuals and their ethnicity, and medical comorbidities, the endometrial susceptibility to the virus could differ based on viral cell entry mechanisms [19]. Although several animal studies have suggested the possibility of inhibition and regression of endometriotic lesions by blocking ACE II type 1 receptor (AT1R) and suggest that activation of peroxisome proliferator-activated receptor (PPAR)-g prevents vascularization and growth in murine model [22, 23]; human studies are required to determine the risk of COVID-19 infection in the endometrial tissue [24].

From the clinical perspective, it is not yet clear whether patients with thoracic endometriosis have a higher risk of pulmonary disease or COVID-19 infection [25]. An expert opinion has suggested specific treatment guidelines, in order to reduce the susceptibility of endometriosis patients to COVID-19 infection [26]. As an inflammatory disease, endometriosis stimulates immune response and inflammation in the body [27], which is also a common feature of COVID-19, during which the overly vigorous immune response can result in cytokine storm [28]. So, we hypothesized that while a patient suffering from this inflammatory disease (endometriosis), the patient can be more susceptible to COVID-19, compared to the normal population. As, to date, there is no evidence about the risk of COVID-19 infections in patients with endometriosis, the present study aimed to compare the risk of COVID-19 between women with and without endometriosis.

## Methods

### Study design

This study was designed as a case–control study and conducted at Pars general hospital from May 21st to July 3rd, 2020. The study population consisted of women with histologic confirmation of endometriosis (extracted sample during laparoscopy), diagnosed at least a year before and maximum of 10 years before the study, compared with an age-matched control group, selected from women without endometriosis who referred to the gynecologic clinic for screening Pap smear test and had no complaints of any symptom related to endometriosis. All participants were asked to complete a researcher-designated checklist via email or social networks or cell phone for evaluation of Real-Time Polymerase Chain Reaction (rt-PCR) screening test and symptoms of COVID-19, a recent history of traveling to the high-risk areas, commitment to social distancing, relationship with a patient infected with COVID-19, positive COVID-19 rt-PCR test, history of isolation due to COVID-19 infection, hospitalization due to COVID-19, H1N1 infection, and vaccination during last year, and other medical diseases. The symptoms asked from patients included fever, sore throat, nasal congestion, cough, shortness of breath, headache, weakness and muscle pain, reduced sense of smell and/or taste, ocular problems, and other (including gastrointestinal, skin, hematologic, and neuronal) complications. The rt-PCR test is performed by Dacron or Rayon swabs taken from naso- or oro-pharyngeal, from any patients with suggestive symptomsDacron or Rayon swabs taken from naso- or oro-pharyngeal, from any patients with suggestive symptoms, perform the rt-PCR test. The samples were sent to the laboratory immediately, where COVID-19 nucleic acid was extracted and used for molecular detection by fluorescence probing. Patients, younger than 18 or older than 45 were excluded from the study.

The sample size of the study was considered at a minimum of 507 in each group. For sample size calculation, we considered the prevalence of COVID-19 reported by Quartuccioa et al. at 3.8; in every 1000 patients with chronic inflammatory disease due to the lack of evidence on COVID-19 in women with endometriosis) (and 2 in every 1000 in general population) [29], using the following equation and considering the power of the study at 80% and alpha error at 0.05.

$$m = \frac{{\left\{ {z_{1 - a/2} \sqrt {\left[ {2\overline{p}\overline{q}} \right]} + z_{1 - \beta } \sqrt {\left[ {p_{1} q_{1} + p_{2} q_{2} } \right]} } \right\}}}{{\delta_{{{\text{plan}}}}^{2} }}$$

The researcher selected the participants according to the inclusion criteria, explained the study design and objectives to the eligible participants, and asked them to read and sign the written informed consent; then, the researcher included the eligible participants (who gave consent) into the study by census method. The protocol of the present study was approved by the Ethics Committee of Pars Advanced and Minimally Invasive Medical Manners research center, Pars Hospital, Tehran, Iran. (Code: 99G5018).

### Statistical analysis

For describing the categorical variables, frequency (percentage) was reported. For numeric variables, first, Kolmogorov–Smirnov test was used to assess the normal distribution of data and according to the results of this test, the numeric variables were described by mean ± standard deviation (SD) or median and compared between the groups using independent *t-test* or Mann–Whitney U test, whenever the data did not appear to have normal distribution or when the assumption of equal variances was violated across the study groups. Categorical variables were, on the other hand, compared using chi-square or Fisher’s exact test. The association of variables was tested by Spearman’s correlation coefficient. For the statistical analysis, the statistical software IBM SPSS Statistics for Windows version 21.0 (IBM Corp. 2012. Armonk, NY: IBM Corp.) was used. *P* values of 0.05 or less were considered statistically significant.

## Results

A total of 507 women were evaluated in the case group and 520 women in the control group. The mean ± SD of the women’s age was 29.08 ± 14.29 in the case group and 33.00 ± 7.06 in the control group (*P* = 0.379). The majority of the case group had stage IV endometriosis (N = 110, 63.2%), 17.2% had stage III (N = 30), 8% had stage II (N = 14), 11.4% had stage I endometriosis (N = 20). In the case group, 18.3% (N = 93) had a positive history of infertility.

The results of comparing the COVID-19 characteristics between the case and control groups, as shown in Table 1, showed no difference between the groups in terms of COVID-19 infection (*P* = 0.942), frequency of H1N1 vaccination, recent traveling to high-risk provinces, social distancing, close contact with an infected patient, as well as the frequency of performing screening test, admission and isolation due to COVID-19 (*P* > 0.05); but, the frequency of symptoms (*P* < 0.05) and H1N1 infection were significantly different between the groups (*P* < 0.001). As shown in Table 1, the frequency of asymptomatic cases and the frequency of fever was higher in the control group (*P* < 0.001 and 0.004, respectively), and the frequency of other symptoms was higher in the case group (*P* < 0.001). The average disease period was 14 days in both groups (*P* = 0.694).

**Table 1:** The results of comparing the coronavirus disease characteristics between the case and control groups

Variable	Categories	Case group (N = 507)		Control group (N = 520)		*p*-value
		Number	Percent	Number	Percent	
H1N1 infection	No	462	91.1	490	2.0	< 0.001^a^
	Yes	44	8.7	10	2.0	
H1N1 vaccine	No	488	96.3	495	97.4	0.212^a^
	Yes	18	3.6	13	2.6	
Travel	No	470	92.7	370	69.7	0.059^a^
	Yes	36	7.1	24	4.5	
Social distancing	No	397	78.3	267	67.8	0.256^a^
	Yes	109	21.5	127	32.2	
Close contact	No	475	93.7	358	91.8	0.979^a^
	Yes	31	6.1	32	8.2	
COVID-19 infection	No	490	96.6	515	97	0.942^a^
	Yes	16	3.2	16	3	
symptoms	None	479	94.5	508	95.7	< 0.001^a^
	Fever	0	0	8	1.6	0.004^b^
	Sore throat	7	1.4	6	1.2	0.745^a^
	Nasal congestion	8	1.6	2	0.4	0.050^a^
	Cough	7	1.4	6	1.2	0.747^a^
	Shortness of breath	8	1.6	6	1.2	0.558^a^
	Headache	4	0.8	3	0.6	0.486^a^
	Weakness and muscle pain	5	1.0	13	2.6	0.094^a^
	Reduced sense of smell and/or taste	9	1.8	7	1.4	0.622^a^
	Ocular problems	4	0.8	1	0.2	0.179^b^
	Other	11	2.2	0	0	< 0.001^b^
Screening	No	477	94.1	476	89.6	0.137^a^
	Yes	29	5.7	42	7.9	
Admission	No	505	99.6	520	100	0.494^b^
	Yes	1	0.2	0	0	
Isolation	No	493	97.2	420	97.2	0.790^a^
	Yes	13	2.6	12	2.8	

Results of: ^a^Chi square test, ^b^Fisher’s exact test

The frequency of underlying diseases is shown in Table 2. As demonstrated in this table, 80.5% in the case group and 72.3% in the control had no underlying disease (*P* = 0.002) and the frequency of diabetes mellitus (*P* = 0.038), cardiovascular disease, hypertension, and lupus erythematosus were higher in the control (all *P* < 0.001; Table 2).

**Table 2:** The results of comparing the frequency of underlying diseases between the study groups

	Case group (N = 507)		Control group (N = 520)		*p*-value
	Frequency	Percent	Frequency	Percent	
None	408	80.5	376	72.3	0.002^a^
Thyroid disease	5	0.98	3	0.57	0.501^b^
Diabetes mellitus	11	2.2	23	4.6	0.038^a^
Cardiovascular disease	2	0.4	36	7.2	< 0.001^a^
Hypertension	13	2.6	42	8.4	< 0.001^a^
Asthma	1	0.2	6	1.2	0.124^b^
Allergy	13	2.6	23	4.6	0.057^a^
Cancer	5	1.0	11	2.2	0.097^a^
Sinusitis	6	1.2	2	0.4	0.173^b^
Lupus erythematosus	2	0.4	6	1.2	0.156^b^
Rheumatoid arthritis	4	0.8	26	5.2	< 0.001^b^
Other	18	3.6	24	4.8	0.314^a^

Results of: ^a^Chi-square test, ^b^Fisher’s exact test

Studying the association of the study variables with COVID-19 infection identified “close contact with a patient infected with COVID-19” as a significant risk factor, both in the case (r = 0.331, *P* < 0.001) and the control group (r = 0.244, *P* < 0.001), while other variables such as social distancing, traveling, underlying diseases, thyroid disease, and endometriosis stage were not associated with COVID-19 infection (*P* > 0.05; Table 3).

**Table 3:** The association of COVID-19 infections with the study variables in each study group

	COVID-19-positive cases in the case group (N = 16)			COVID-19-positive cases in the control group (N = 16)		
	N (%)	Pearson’s coefficient	*p* value	N (%)	Pearson’s coefficient	*p* value
*Underlying diseases*						
Diabetes mellitus	–	0.108	0.611	1 (6.2%)	0.202	0.533
Cardiovascular disease	–			1 (6.2%)		
Hypertension	–			2 (12.5%)		
Asthma	–			1 (6.2%)		
Allergy	–			1 (6.2%)		
Rheumatoid arthritis	2 (12.5%)			1 (6.2%)		
Thyroid disease	5 (31%)	0.032	0.471	3 (18.6%)	0.026	0.588
Admission due to COVID-19	1 (6.2%)	0.246	< 0.001	0	–	–
H1N1 vaccination	1 (6.2%)	0.026	0.445	13 (81.2%)	0.026	0.554
Travel	1 (6.2%)	0.006	1.000	4 (25%)	0.803	0.465
Social distancing	5 (31%)	0.043	0.355	4 (25%)	0.089	0.510
Close contact	8 (50%)	0.331	< 0.001	6 (37.5%)	0.244	< 0.001

## Discussion

Comparing two groups of women with and without endometriosis showed no difference in the frequency of COVID-19 infection, which is contrary to the initial hypothesis of our study, as we assumed that patients with endometriosis, an inflammatory disease, have a greater susceptibility to COVID-19 infection, because of their baseline disorder in the immune system, as well as the evidence on the expression of ACE_2_ in the endometrial tissue [15, 18]. Studies have reported different diseases that can predispose the individual to COVID-19 and reported that malnutrition [30], serum parameters, such as blood group [31], and elevated plasmin(ogen) [32], as well as underlying autoimmune diseases, such as tuberculosis [33] and lupus erythematosus [34] can increase patients’ susceptibility to COVID-19. However, as far as the authors are concerned, the risk of COVID-19 infection in women with endometriosis has not been clinically evaluated, to date.

The endometrial susceptibility to COVID-19 is still under investigation. Molecular investigations have shown that ACE_2_ is expressed in endometrial epithelial cells and stromal cells in the proliferative phase of the menstrual cycle, which increases in the secretory phase and affects the in vivo decidualization, embryo implantation, and placentation [18]. The expression of ACE_2_ has been also confirmed in the myometrium and uterine leiomyoma [15]. In another molecular genetic study, it was demonstrated that the lower expression of host proteases, related to SARS-CoV-2 infection, such as ACE_2_ and TMPRSS2 may result in a lower risk of endometrial susceptibility to COVID-19 infection, but the expression of these proteins varies in different phases of the menstrual cycle and increases during implantation and in older women [19]. It is also assumed that COVID-19 can induce changes in endometrial tissue and affect the female reproductive system [12]. However, the studies available in this regard are expert opinion or molecular based and the susceptibility of endometrial tissue to COVID-19 has not been confirmed in the clinical setting [24].

It has been previously demonstrated that despite the indefinite pathophysiology of endometriosis, the immune system is considered as a cause of development of endometriosis and several immunologic and inflammatory changes are observed in endometriosis [35]. The main immunologic changes in endometriotic patients include reduction of T cell reactivity, natural killer (NK) cell’s cytotoxicity, increased antibody production, macrophages polarization and augmented release of inflammatory disease [36]. The increased infiltration level of immune cells, including B cell, CD4^+^T cell, neutrophil, and dendritic cells as well as increased expression of ACE_2_ has been correlated with SARS-CoV-2 susceptibility in endometrial cancer [31]. However, such association has not been found in endometriosis and the results of our study showed no difference in susceptibility to COVID-19 disease in women with endometriosis, the underlying reason requires further investigations.

In the current study, it was found that the frequency of COVID-19 symptoms differed between women with and without endometriosis; endometriotic women had a lower frequency of asymptomatic and febrile infection, but higher frequency of other symptoms, including gastrointestinal, dermatologic, hematologic, and neuronal disorders. Keeping in mind that the difference in the symptoms may, also, be related to the difference in the underlying diseases of the patients and the medications they use [37], our results suggest that more attention should be paid to women with endometriosis for diagnosis of COVID-19 infection, as they mainly do not present common symptoms, which can make diagnosis difficult [38]. COVID-19 infection interferes with the antigen-presenting cells in the immune system and creates bilayer vesicles, which can block the expression of Pattern Recognition Receptor (PRR) and, as a result, the patient’s innate immune system does not recognize them and continue to proliferate within the vesicle, they also, disable the production of Type I interferon as one of the most important antiviral factors so it will develop as an asymptomatic disease in some cases [39]. Asymptomatic COVID-19 is considered the Achilles’ heel for disease control, due to the strong infectivity and transmission during this period, and the major role of asymptomatic carriers in the person-to-person disease transmission [40]. We suppose that the different clinical presentation of COVID-19 in women with endometriosis in the present study can be attributed to the altered immune interactions in endometriosis [41, 42], similar to the different disease characteristics of COVID-19 in other immune-mediated inflammatory diseases/conditions, such as rheumatoid arthritis, psoriatic arthritis, ankylosing spondylitis, psoriasis, inflammatory bowel disease [43], and pregnancy [44]. Further molecular studies are required to understand the exact mechanism of this finding.

We also analyzed factors associated with COVID-19 infection and the results revealed that close contact with a patient infected with COVID-19 was the only risk factor in both groups that resulted in a slightly increased chance (0.3- and 0.2-folds higher odds in the case and control groups, respectively), while other variables such as social distancing, traveling, underlying diseases, thyroid disorders, and endometriosis stage were not associated with COVID-19 infection. As suggested, preventive measures should be considered by everyone to reduce contact with an infected person (possibly in the incubation or asymptomatic period) to reduce the transmission rate and the prevalence of this epidemic [45, 46], which requires increasing the knowledge and awareness of the general population about the necessary precautions to be taken during the current outbreak [47].

The limitations of the present study include the cross-sectional nature of the study and lack of follow-up. Therefore, we could only suggest associations, rather than the causal relationship between the study variables. Furthermore, we matched the control group in terms of age with the case group and selected women were from the same medical center; however, differences in other characteristics between the groups may affect the results. Also, we recruited participants by census method and the nonrandomized patient selection increases the chance of confounders on the results. For sample size calculation, we could not use the statistics of our own region, as published data was not available at the time of sample size calculation and we had to estimate the real prevalence based on the available evidence.

## Conclusions

The results of the present study suggested that endometriosis does not increase the susceptibility to COVID-19 infection, but it changes the presenting symptoms; although more homogenous groups are required for definite conclusions. It is thus suggested to pay more attention for accurate diagnosis of COVID-19 in women with endometriosis. Since the exact mechanism of infection with this virus in these patients is not fully understood, the most important task at present is to eliminate the transmission cycle, for which identifying the predisposing factors can help diagnose high-risk patients.

## Data Availability

The corresponding author, Shahla Chaichian, can be contacted if someone wants to request the data.

## Abbreviations

COVID-19 infection: Coronavirus disease 2019
ARDS: Acute respiratory distress syndrome
ICU: Intensive care unit
MOF: Multiple organ failure
ACE_2_: Angiotensin-converting enzyme 2
TMPRSS2: Transmembrane protease serine protease-2
rt-PCR: Real-time polymerase chain reaction
SD: Standard deviation
NK: Natural killer
PRR: Pattern recognition receptor
CT: Computed tomography
